# Serratia Sacroiliitis Secondary to Intravenous Drug Use: A Case Report

**DOI:** 10.7759/cureus.67683

**Published:** 2024-08-24

**Authors:** Blake E Delgadillo, Zachary J Buchman, Kassidy Webber, Justin R Federico

**Affiliations:** 1 Department of Orthopedic Surgery, Lake Erie College of Osteopathic Medicine, Bradenton, USA; 2 Department of Internal Medicine, Baptist Health, Jacksonville, USA

**Keywords:** si joint, sacroiliac osteomyelitis serratia marcescens, osteomyelitis, sacroiliac septic arthritis, septic sacroiliitis, serratia marcescens, serratia osteomyelitis, serratia, septic arthritis, intravenous drug use (ivdu)

## Abstract

*Serratia *spp. are ubiquitous, opportunistic, and infectious organisms that have historically been known to infect the upper respiratory, urinary, and circulatory systems. This manuscript presents the case of a 35-year-old White female with a past medical history of polysubstance abuse, intravenous drug use (IVDU), and poor dentition who was admitted to a community hospital with complaints of lower back pain for 10 days following the recent completion of treatment for a suspected epidural abscess. Per her report, her last IVDU with fentanyl was 11 days prior, and she admitted to using various sources of water to inject her drugs. Magnetic resonance imaging with contrast was significant for possible infectious sacroiliitis, and blood cultures grew *Serratia marcescens*. Due to this patient's extensive IVDU history, in-patient ceftriaxone was chosen over discharging the patient with a peripherally inserted central catheter line. *Serratia *spp.bacteremia with concomitant septic sacroiliitis in the setting of IVDU is an extremely rare presentation. Due to the nonspecific presentation of sacroiliitis, multidrug resistance profile of *Serratia *spp., and high mortality rate associated with *S. marcescens *sepsis, early detection and diagnosis is paramount in similar patients with extensive risk factors.

## Introduction

Roughly one-quarter of all cases of lower back pain can be attributed to pathology involving the sacroiliac (SI) joint, which is a highly stable joint with very limited motion [1]. These qualities make the SI joint a peculiar location for joint pain, with many cases occurring in younger patients due to sports injuries or pregnancy or in older patients due to joint degeneration [1]. Other causes of SI joint pain include trauma, prior lumbosacral fusion surgery, ankylosing spondylitis, scoliosis, and joint infection [1,2]. Sacroiliitis, defined as the inflammation of one or both SI joints, can occur due to a few of the aforementioned causes of SI joint discomfort and often results in additional pain involving the leg(s) [1]. Treatment of the condition is based on the underlying etiology of inflammation, which may be rheumatologic, mechanical, or infectious. Of these causes, infection is the least common, with only 1.5%-10% of SI conditions being attributed to infection [3]. When considering septic arthritis and osteomyelitis as a whole, SI joint involvement is virtually just as rare as it occurs in as few as 1% of cases [3]. As a result, infection involving the SI joint can often be overlooked and heavily reliant on the presence of other diagnostic indicators, such as specific imaging and laboratory findings.

Factors that place a patient at risk of developing osteomyelitis include bacteremia, endocarditis, intravenous drug use (IVDU), trauma, open fractures, diabetes, peripheral vascular disease, peripheral neuropathy, and previous placement of orthopedic hardware [4]. Knowledge of these risk factors is essential because the presentation of osteomyelitis is somewhat nonspecific and may or may not include fever, chills, dull pain in the area, generalized malaise, arthritic pain, and cardinal signs of inflammation surrounding the area of infection [5]. Laboratory data showing leukocytosis and elevation of other inflammatory markers, such as erythrocyte sedimentation rate (ESR) and C-reactive protein (CRP), should increase clinical suspicion of osteomyelitis in the setting of the aforementioned symptoms, and this presentation should prompt blood culture and imaging to confirm the diagnosis [5]. Imaging is crucial in diagnosing osteomyelitis, and magnetic resonance imaging (MRI) has the highest sensitivity and specificity for detection [6,7]. With the ability to detect osteomyelitis after only three to five days, an MRI can detect signs of this infection roughly 10 days sooner than can be detected using an X-ray [6,7]. Without detection and adequate treatment of osteomyelitis, complications such as septic arthritis, pathological fractures, squamous cell carcinoma, sinus tract formation, abscess, bone deformity, systemic infection, or even death can result [5].

Treatment for osteomyelitis is tailored to the specific microbial origin of infection, considering strain-specific antibiotic susceptibility. Although rates vary depending on the causative microbe(s), the mortality rate for osteomyelitis due to *Staphylococcus aureus *is 8%, which is nearly one-third that of *Serratia marcescens* (22.4%) [8,9]. *S. marcescens *is a ubiquitous, opportunistic, and infectious organism that has historically been known to infect the upper respiratory, urinary, and circulatory systems [10]. Of note, this gram-negative bacillus has been reported to cause infective endocarditis, particularly in IVDU patients [10]. In hospitals, *Serratia *spp. pose a particular threat as nosocomial isolates are typically multidrug-resistant and have been shown to have the capacity to synthesize lipopolysaccharide, hemolysin, and siderophores, which often results in a difficult treatment course [11,12]. Apart from its typical infective locations, there have been minimal reported cases of *Serratia *spp.osteomyelitis, with even less of it, contained in the axial skeleton, which creates a challenge for any diagnostician [13]. There is only one other reported case of sacroiliitis due to *S. marcescens*, and it involves a patient with IVDU and hepatitis C [14]. This case of *Serratia* sacroiliitis secondary to IVDU highlights the importance of recognition of the risk factors for its development, imaging findings, and early intervention due to its easily missed diagnosis and grave consequences for the patient if such a mistake is made.

## Case presentation

**Figure 1:**
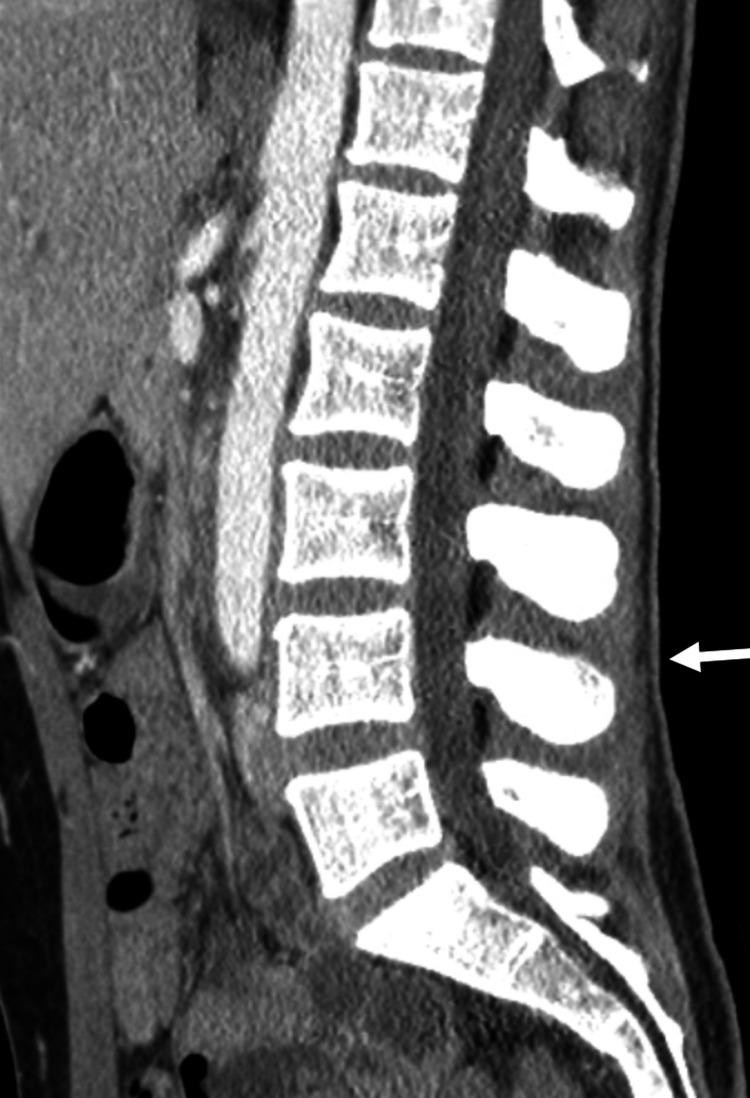
Sagittal lumbar spine computed tomography with contrast, showing nonspecific mild induration of the subcutaneous fat overlying the L3/L4 spinous processes (white arrow)

**Figure 2:**
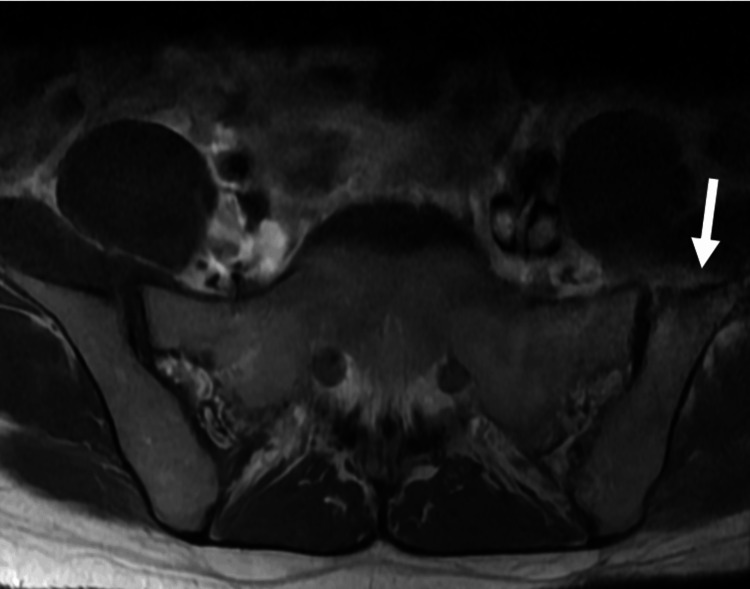
Transverse lumbar spine MRI with contrast, demonstrating a mild T1 hypointense marrow signal along the periphery of the left SI joint with possible joint erosion (white arrow) MRI: magnetic resonance imaging; SI: sacroiliac

**Figure 3:**
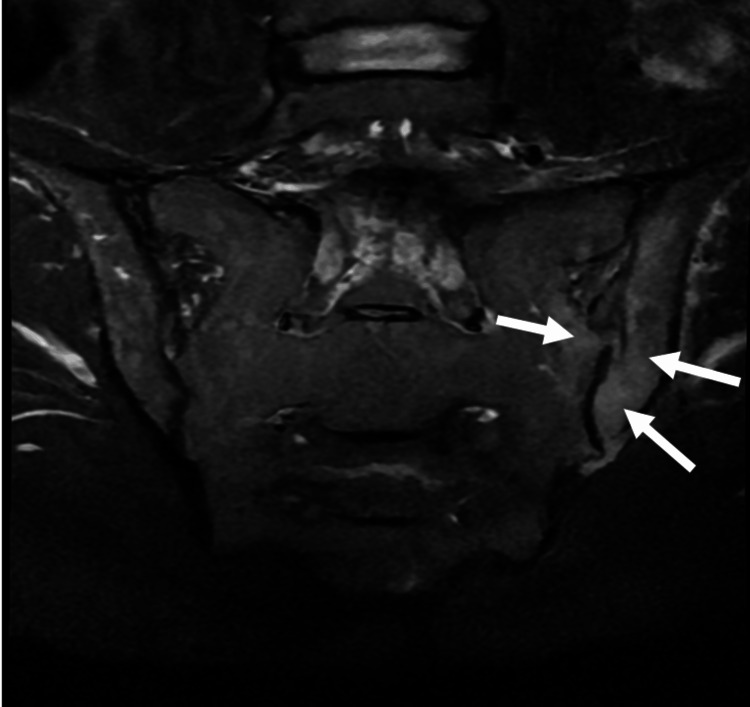
Coronal sacrum and coccyx MRI with contrast, showing an increased T2 signal within the left SI joint and adjacent osseous structures (white arrows) MRI: magnetic resonance imaging; SI: sacroiliac

A 35-year-old White female with a past medical history of polysubstance abuse, IVDU, and poor dentition was admitted to a community hospital with complaints of lower back pain radiating to the left lower leg, pain with movement, and progressive inability to ambulate for 10 days following recent completion of treatment for a suspected, although not definitively diagnosed, epidural abscess. She denied numbness, urinary or fecal incontinence, dysuria, hematuria, and abdominal pain. She reported that her last IVDU was 11 days prior, and she admitted to occasionally using tap water to dilute her injections. Computed tomography of the lumbar spine showed mild induration of the subcutaneous fat overlying the L3/4 spinous process, which is nonspecific but could be related to a subcutaneous infection (Figure 1). An MRI with contrast was ordered due to her increasing back pain and developing radicular symptoms, which showed a T1 hypointense marrow signal along the periphery of the left SI joint, a possible left SI erosion, and signal enhancement immediately anterior to the left SI joint, which indicates a potentially infectious sacroiliitis (Figures 2, 3). Laboratory results revealed an elevated white blood cell count of 14.70/µL and mild anemia with a hemoglobin level of 10.9 g/dL. Routine laboratory tests were otherwise unremarkable. Empiric antibiotics, consisting of vancomycin and piperacillin/tazobactam, opioid pain control, antiemetics, and deep vein thrombosis prophylaxis, were given. The patient met sepsis criteria with tachycardia and leukocytosis. Hepatitis C was found to be positive, but hepatitis B and human immunodeficiency virus were negative. Blood cultures grew *S. marcescens*, a gram-negative bacillus. Neurosurgery was consulted, who advised medical treatment. Due to her extensive IVDU history, discharging her with a peripherally inserted central catheter line was ill-advised. Therefore, over the following weeks, the infectious disease team transitioned her antibiotics to ceftriaxone for planned inpatient intravenous (IV) administration.

## Discussion

Osteomyelitis is much more common in areas of the body such as the vertebral bodies (most common in adults), long bones, and clavicles [4,15]. This infection requires special circumstances to occur, as healthy bone is generally resistant to disease. For example, possible instances may include the introduction of a significant bacterial load, trauma, ischemia, or the presence of foreign bodies acting as a nidus for infection [16]. Common mechanisms for developing osteomyelitis include hematogenous inoculation through bacteremic seeding, contiguous spread from nearby tissue, or direct inoculation of bone from trauma or surgery [4]. Furthermore, depending on the location within a bone, septic arthritis can also result if the infection extends into part of the bone within a joint capsule [5]. Due to these etiologies, it is logical that the risk of developing osteomyelitis is increased by bacteremia, endocarditis, IVDU, trauma, open fractures, diabetes, peripheral vascular disease, peripheral neuropathy, and previous placement of orthopedic hardware [4].

These risk factors, specifically bacteremia, extend beyond solely being a risk factor and may also serve as a possible etiology for osteomyelitis. In the case of this patient, without trauma or recent surgery, hematogenous seeding of *S. marcescens* to the sacrum/ilium serves as a strong possibility for the root cause of this patient's sacroiliitis. Interestingly, risk factors for bacteremia specifically due to *S. marcescens* include long-term immunosuppressive therapy, prior antibiotic use, indwelling catheterization, and underlying chronic disease [17]. Considering the rarity of osteomyelitis due to this bacterium, it was expected that the rare patient with this condition would have more than one risk factor (prior antibiotic use to treat a spinal abscess less than two weeks before this presentation) for developing bacteremia due to *S. marcescens*. In fact, in the only other reported case of sacroiliitis due to *S. marcescens*, the patient had a much more extensive medical history in addition to IVDU, including Hepatitis A and a previous instance of bacteremia [14]. Nevertheless, this other case bears a striking resemblance, as the patient was relatively young with a prior history of IVDU and hepatitis C. The patient complained of lower back pain, difficulty bearing weight, and lumbar radicular symptoms [14]. The patient presented in this manuscript was treated with IV ceftriaxone, but in the report by Simon et al. [14], the patient received IV ceftriaxone and IV vancomycin.

For patients with an incompletely documented history of IVDU, an extensive discussion should take place as infectious sources and presentations can be highly variable and difficult to identify. This is especially true in patients who present with radicular symptoms and/or trouble ambulating as a less benign cause of the pain may be the culprit, as it was in this case and the study by Simon et al. [14]. Without considering the history of IVDU and possible spinal abscess, a patient with back pain described by the patient may have been dealt with in a much more conservative manner or with the perspective of possible malingering. In this scenario, failure to act would not reveal the patient's leukocytosis, qualification for sepsis criteria, and a serious etiology of her lower back pain [18]. Such an unfortunate occurrence would impair prompt diagnosis and treatment, which is vital to the health of affected patients, as untreated *S. marcescens* sepsis has high mortality rates, which can be exacerbated in patients with concurrent risk factors [9].

## Conclusions

*Serratia *spp. are not an uncommon source of bacteremia, though concomitant septic sacroiliitis in the setting of IVDU is an extremely rare presentation. Nonetheless, grave consequences can occur if the diagnosis is missed. Recognition of the risk factors to develop osteomyelitis due to *Serratia *spp., along with signs of atypical back pain, especially in someone with IVDU, should spur a workup including complete blood count, ESR, CRP, MRI with or without X-ray, as well as blood cultures. Due to the nonspecific presentation of sacroiliitis, multidrug resistance profile of *Serratia *spp., and high mortality rate associated with *S. marcescens* sepsis, early detection and diagnosis are paramount in similar patients with extensive risk factors. This manuscript serves as just a second example of a dangerous case of SI osteomyelitis due to *S. marcescens* and provides a reference for suspected cases of such in patients with a history of IVDU complaining of sacroiliitis.
